# Supplementary material for: Seminoma and Embryonal Carcinoma Footprints Identified by Analysis of Integrated Genome-Wide Epigenetic and Expression Profiles of Germ Cell Cancer Cell Lines
Source: PLoS One. 2014 Jun 2;9(6):e98330. doi: 10.1371/journal.pone.0098330 (PMC4041891; doi:10.1371/journal.pone.0098330)
Supplement: File S1 — ZIP file containing DMRforPairs output for significant regions. Please start from the html files. (ZIP) [file pone.0098330.s008.zip › figures/11639.pdf]

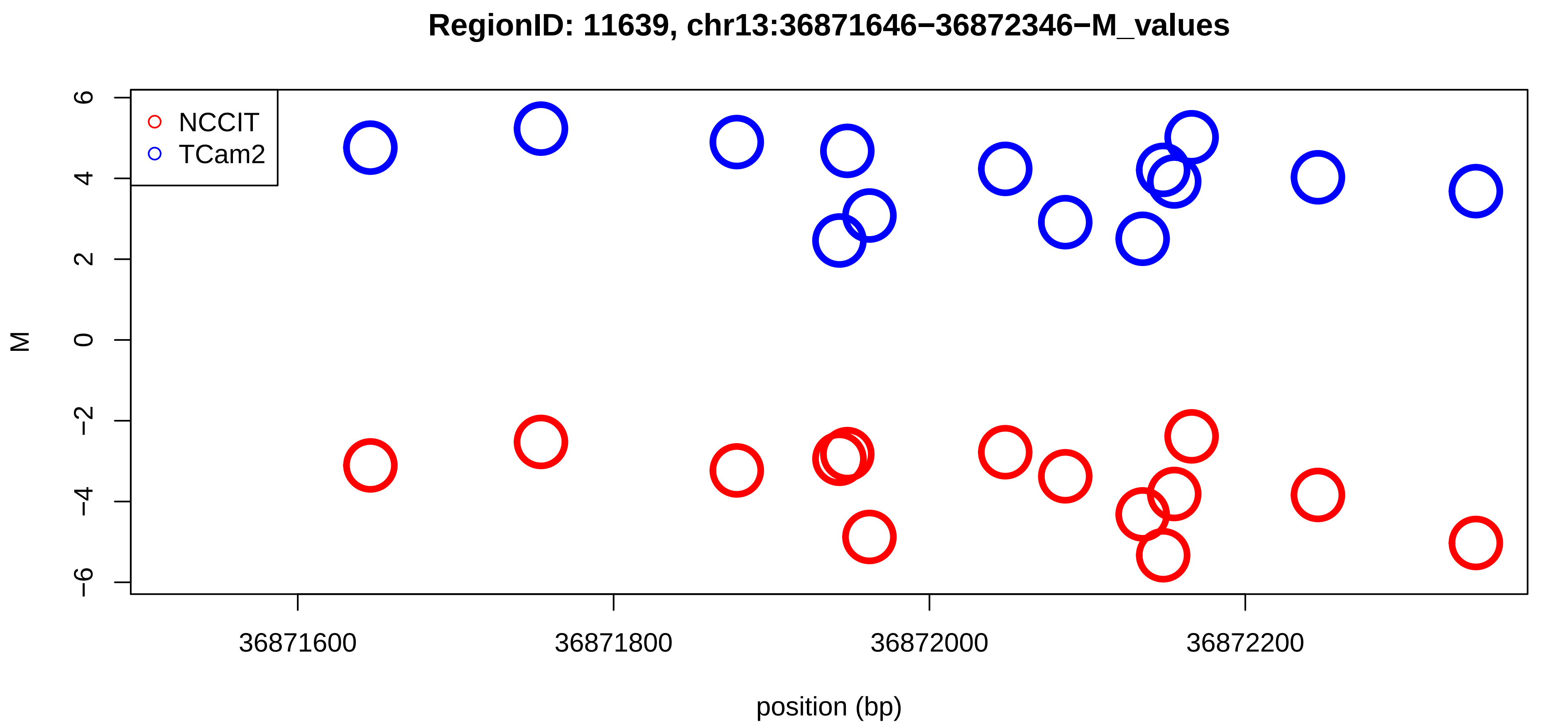
RegionID: 11639, chr13:36871646−36872346−M_values
NCCIT
TCam2
M
6
4
2
0
−2
−4
−6
36871600
36871800
36872000
36872200
position (bp)

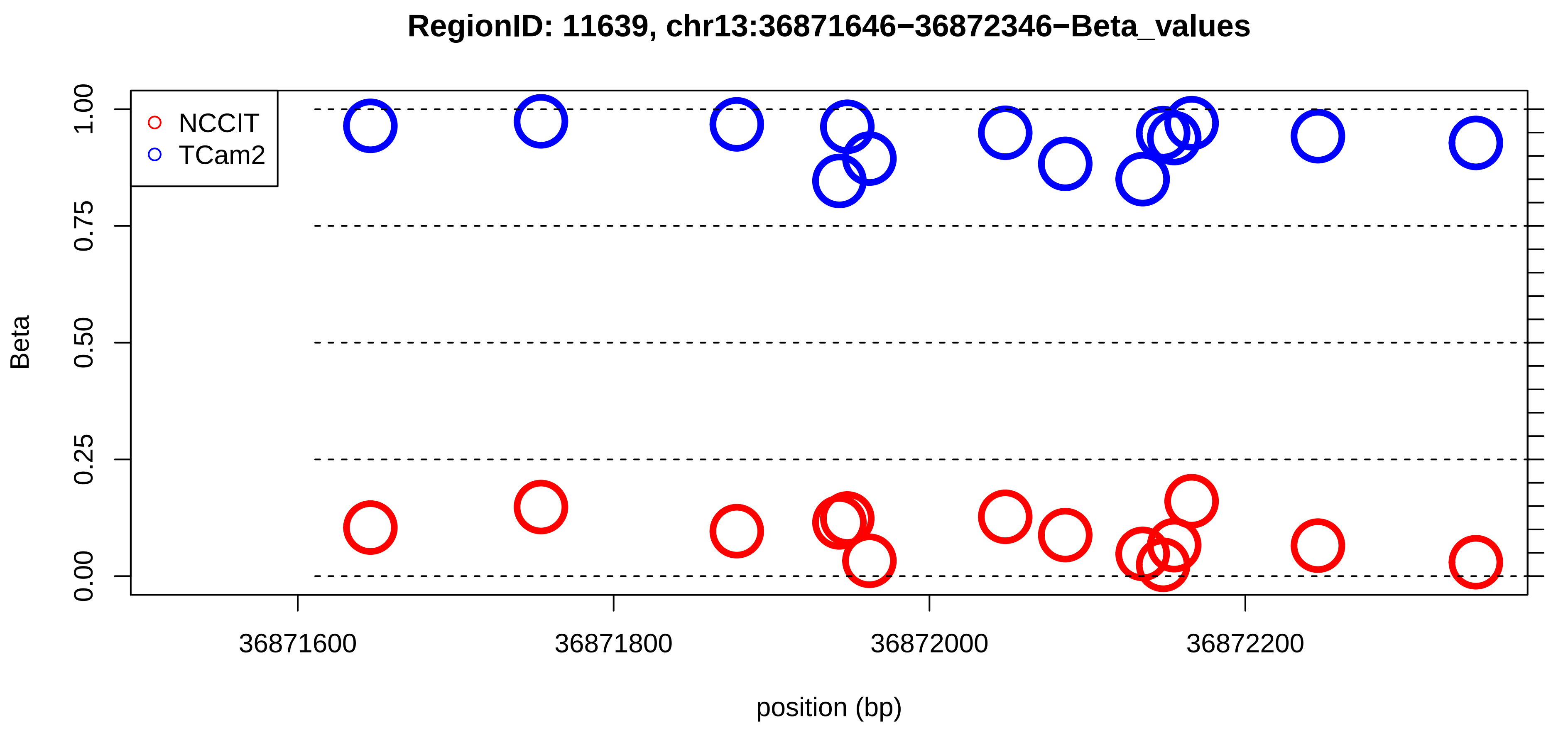
RegionID: 11639, chr13:36871646−36872346−Beta_values
NCCIT
TCam2
Beta
1.00
0.75
0.50
0.25
0.00
36871600
36871800
36872000
36872200
position (bp)

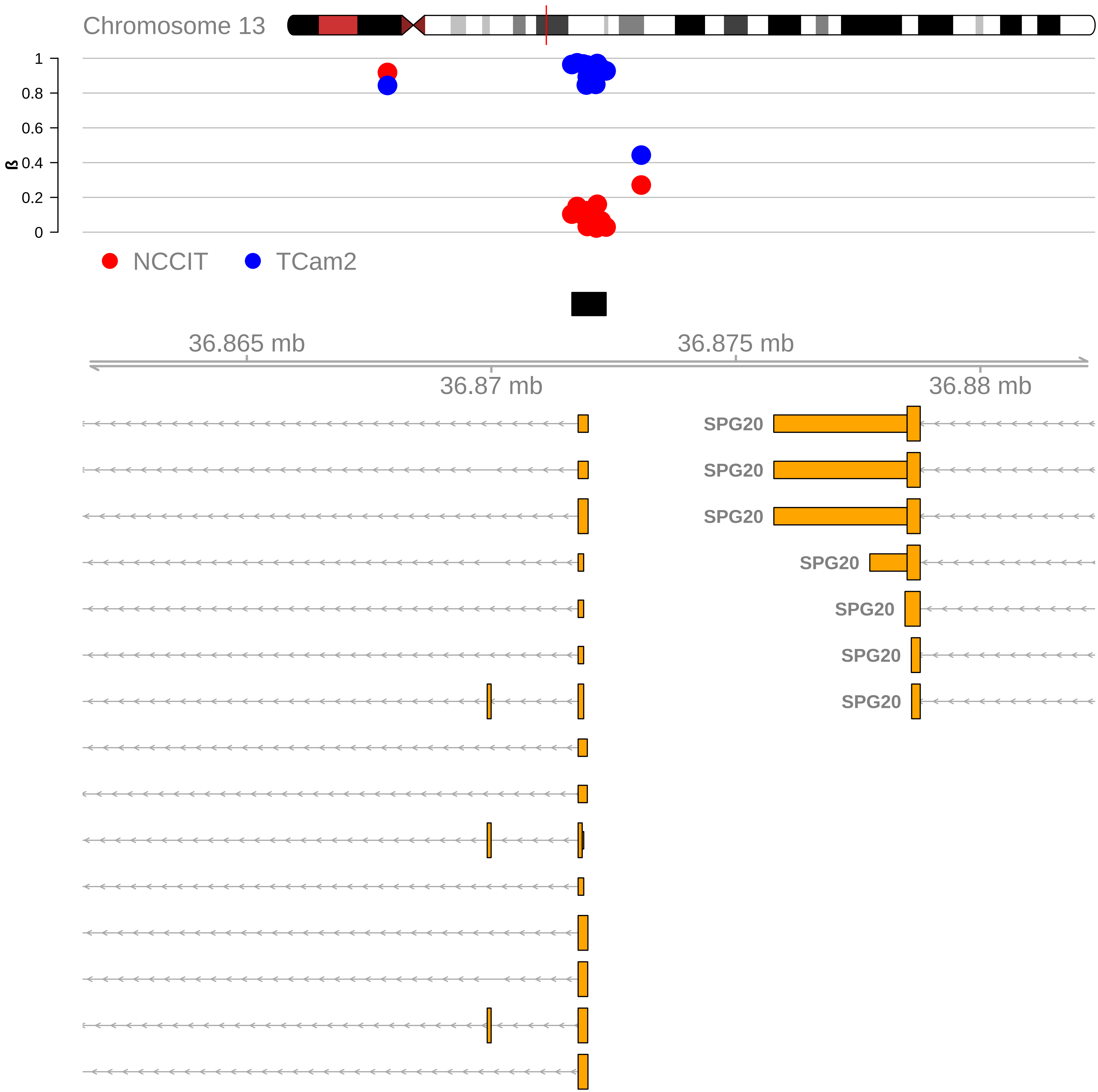
Chromosome 13
1
0.8
0.6
0.4
0.2
0
β
NCCIT
TCam2
36.865 mb
36.875 mb
36.87 mb
36.88 mb
SPG20
SPG20
SPG20
SPG20
SPG20
SPG20
SPG20
